# Systemic immune-inflammation index is associated with diabetic kidney disease in Type 2 diabetes mellitus patients: Evidence from NHANES 2011-2018

**DOI:** 10.3389/fendo.2022.1071465

**Published:** 2022-12-06

**Authors:** Wencong Guo, Yancheng Song, Yan Sun, Huasheng Du, Yan Cai, Qingqing You, Haixia Fu, Leping Shao

**Affiliations:** ^1^ Laboratory of Nephrology & Department of Nephrology, The Affiliated Qingdao Municipal Hospital of Qingdao University, Qingdao, Shandong, China; ^2^ Department of Gastrointestinal Surgery, The Affiliated Hospital of Qingdao University, Qingdao, Shandong, China

**Keywords:** systemic immune-inflammation index, type 2 diabetes mellitus, diabetic kidney disease, population-based study, NHANES

## Abstract

**Objective:**

Diabetic kidney disease (DKD) is the most common chronic kidney disease (CKD) and has the highest prevalence of end-stage kidney disease (ESKD) globally, owing mostly to the rise in Type 2 diabetes mellitus (T2DM) correlated with obesity. Current research suggested that the immune response and inflammation may play a role in the pathophysiology of T2DM. The systemic immune-inflammation index (SII) is a novel and integrated inflammatory biomarker that has not yet been linked to DKD. We aimed to identify the potential relationship between SII and DKD.

**Methods:**

In the National Health and Nutrition Examination Survey (NHANES) between 2011 and 2018, the current cross-sectional study was conducted among adults with T2DM. SII was calculated as the platelet count × neutrophil count/lymphocyte count. DKD was diagnosed with impaired glomerular filtration rate (< 60 mL/min/1.73 m^2^ assessed by using the Chronic Kidney Disease Epidemiology Collaboration algorithm), albuminuria (urine albumin to creatinine ratio ≥ 30 mg/g), or both in T2DM patients. To investigate the independent association between SII and DKD, weighted univariate and multivariable logistic regression analyses and subgroup analyses were performed.

**Results:**

The study involved 3937 patients in total, of whom 1510 (38.4%) had DKD for the diagnosis. After adjustment for covariates, multivariable logistic regression revealed that a high SII level was associated with increased likelihood of DKD (OR = 1.42, 95% CI: 1.10-1.83, P = 0.01). Subgroup analyses and interaction tests revealed that age, gender, estimated glomerular filtration rate (eGFR), urine albumin-to-creatinine ratio (ACR), body mass index (BMI), hypertension, hyperlipidemia, anti-inflammation therapy (yes or no), metformin use (yes or no), and insulin use (yes or no) had no significant dependence on this positive relationship (all p for interaction >0.05).

**Conclusions:**

Our results indicate that the higher SII level is associated with DKD in T2DM patients. The SII could be a cost-effective and straightforward approach to detecting DKD. This needs to be verified in further prospective investigations.

## Introduction

Since the increased prevalence of Type 2 diabetes mellitus (T2DM) associated with obesity in recent decades, diabetic kidney disease (DKD) has been the most common chronic kidney disease (CKD) and the leading cause of end-stage kidney disease (ESKD), accounting for over 50% of all cases of ESKD globally (1–3). Besides, Miller RG et al. reported that patients with DKD may have an elevated risk of cardiovascular disease, even if in the early stages of DKD (4). Recent investigations have revealed that individuals with DKD are more at risk of dying after catching COVID-19 (5). According to the prospective multicenter Italian COVOCA study, DM and CKD were closely related to intensive care admission and poor prognosis in patients hospitalized for COVID-19 (6). Thus, to avoid the progression of DKD, early intervention is required. A comprehensive understanding of the potential factors associated with the progression of DKD is essential for establishing effective therapeutic strategies to prevent the onset and progression of DKD in clinical practice.

Previous research has shown that many factors, such as metabolic disturbances and hemodynamic abnormalities induced by hyperglycemia and insulin resistance (IR), play an important role in the progression of DKD (7). Furthermore, IR is associated with a low degree of chronic inflammation and several mediators such as interleukin-1, interleukin-6 and tumor necrosis factor-α. Recent studies demonstrate that the pathophysiology of DKD is multifaceted and DKD has been characterized as a metabolic-driven immunological illness (8). According to current research, both systemic and local renal inflammation play essential roles in the progression of DKD (9). There are many novel pro-inflammatory signaling pathways in the development of DKD, such as inflammasome activation, mitochondrial DNA (mtDNA) release, the nuclear factor kappa B (NF-κB) signaling pathway, toll-like receptors (TLRs), myeloid differentiation primary response 88 (TLRs/MyD88) signaling pathway, adenosine 5′-monophosphate-activated protein kinase (AMPK) signaling pathways, and the hypoxia-inducible factor-1 (HIF) signaling pathway (10). Inflammation is also associated with an increase in reactive oxygen species (ROS) production, leading to mitochondrial dysfunction which triggers beta-cell damage and diabetes worsening (11). Moreover, the microarray analysis has also revealed that the expression of pro-inflammatory genes was significantly elevated in animal models or patients with DKD (12, 13). In addition, some clinical trials discovered that non-steroidal selective mineralocorticoid receptor antagonists (MRA) might slow the development of DKD by decreasing inflammation (14). Overall, these findings clearly demonstrate that inflammation is a vital factor in the progression of DKD.

Numerous studies have demonstrated that inflammation contributes to the deterioration of kidney function. The high-sensitivity C-reactive protein (hs-CRP) is a systemic inflammatory marker that has been associated with the progression of DKD in T2DM patients (15, 16). According to Shankar et al. (17), inflammatory biomarkers (white blood cell count, interleukin-6, hs-CRP, and tumor necrosis factor-α receptor 2) were found to be positively correlated with the results of prevalent CKD. Increases in inflammatory blood cell variables such as procalcitonin (PCT) (18), monocyte-to-lymphocyte ratio (19), and platelet-to-lymphocyte ratio (20) serve as basic indicators of inflammation and have been tested for their capacity to predict CKD. A cross-sectional analysis revealed that neutrophil count is an independent risk factor for CKD development in diabetic individuals (21). However, because these indicators include just one or two kinds of immune-inflammatory cells, they may not adequately reflect the state of inflammation.

The systemic immune-inflammation index (SII) is an integrated and innovative inflammatory marker, and was calculated by platelet count × neutrophil count/lymphocyte count. The SII index was originally used to estimate the prognosis of patients with hepatocellular carcinoma (HCC) by Hu et al. (22), it was then used to predict prognosis in other tumors, such as small cell lung cancer (23), epithelial ovarian cancer (24), esophageal cancer (25), colorectal cancer (26), and cervical cancer (27). SII is now thought to precisely assess inflammation status. SII was independently associated with an increased risk factor for protein energy loss in patients receiving maintenance hemodialysis (28). Additionally, higher SII is associated with an increased risk of T2DM depression (29), disease activity in ulcerative colitis patients (30), peripheral arterial disease (31), urinary albumin excretion (32), testosterone deficiency (33), and osteoporosis in postmenopausal women (34).

The traditional strategies for prevention and treatment of DKD include management of hyperglycemia, hypertension, and hyperlipidemia. For patients with T2DM, lifestyle changes and metformin remain the first-line treatments. With breakthroughs in research on the pathophysiology of DKD, some novel treatments targeting renal inflammation, fibrosis, and oxidative stress have gradually entered clinical practice. Actually, some drugs that are beneficial in alleviating the progression of DKD have anti-inflammatory properties. The drugs of anti-inflammation therapy in T2DM patients include metformin, angiotensin-converting enzyme inhibitors (ACEI), angiotensin II receptor blockers (ARB), sodium-glucose cotransporter 2 inhibitor (SGLT2i), dipeptidyl peptidase-4 inhibitor (DDP-4i), glucagon-like peptide 1 receptor agonists (GLP-1RA), nonsteroidal selective MRA finerenone, etc (10, 35–39). On the contrary, a population-based study suggested that insulin therapy was significantly associated with increased likelihood of DKD in patients with T2DM and high IR (40). Insulin treatment has also been found to not provide a significant decrease in inflammatory markers when compared to metformin or placebo in T2DM patients (41). Therefore, the over-insulinization predisposes to inflammation.

Inflammation has been attributed to kidney damage. However, the role of SII in T2DM patients with DKD remains unclear. We hypothesized that a higher SII would be associated with an increased likelihood of DKD in T2DM patients. Therefore, the purpose of our study was to investigate the relationship between SII and DKD among T2DM patients in the National Health and Nutrition Examination Survey (NHANES) in the United States and determine the value of SII and DKD.

## Subjects and methods

### Data and sample sources

Data were downloaded from the NHANES, a nationally representative cross-sectional survey designed and conducted by the National Center for Health Statistics (NCHS). The survey samples the U.S. population using a stratified, multistage probability approach and offers health and nutrition statistics on the non-institutionalized civilian population in the United States. The NCHS Research Ethics Review Board authorized the survey, verifying that all participants provided informed consent. Detailed statistics are accessible at https://www.cdc.gov/nchs/nhanes/.

**Figure 1:**
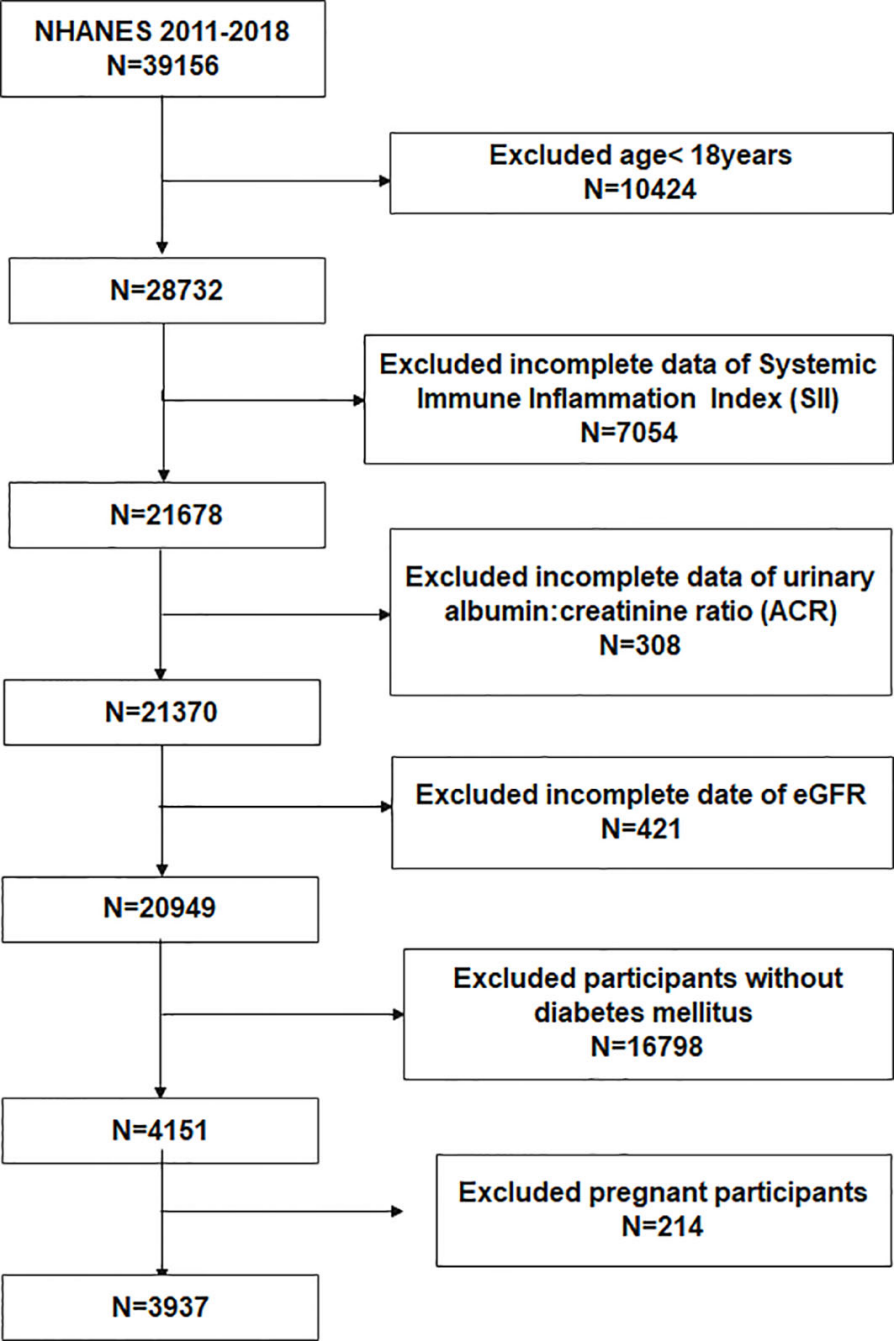
Flowchart of the participants selection from NHANES 2011–2018.

To evaluate the participants’ nutritional and physical health, standardized in-home interviews, physical examinations, and laboratory tests were carried out at mobile examination centers. 39156 participants were involved in four NHANES cycles from 2011-2018. We excluded 10424 participants under the age of 18 years, 7054 with missing SII, 308 without urine albumin-to-creatinine ratio (ACR), 421 with missing estimated glomerular filtration rate (eGFR), 16798 without T2DM, and 214 with pregnancy. Eventually, 3937 participants were enrolled in the study (**Figure 1**).

### Exposure variable

Lymphocyte, neutrophil, and platelet counts (expressed as ×10^3^ cells/μl) were measured using automated hematology analysis devices. The following formula is utilized to calculate SII (22).


$$\text{SII }=\;\frac{\text{platelet count }\times \text{ neutrophil count }}{\text{lymphocyte count}}$$

### Outcome variable

Diabetes was defined as (1) a previously reported diagnosis by medical professionals, or (2) fasting plasma glucose ≥7.0 mmol/L, or (3) glycosylated hemoglobin (HbA1c) ≥6.5 mmol/L, or (4) taking diabetes drugs. The urine albumin/creatinine ratio was used to compute the ACR. The eGFR scores were calculated using the Chronic Kidney Disease Epidemiology Collaboration algorithm (42). ACR ≥30 mg/g and/or eGFR<60 mL/min/1.73m^2^ were used to diagnose DKD in T2DM patients (43).

### Definition of other variables

Hypertension was defined as SBP ≥140 mmHg and/or DBP ≥90mmHg after repeated examination or prior diagnosis of hypertension by a physician (44). Hyperlipidemia was defined by total cholesterol ≥240 mg/dL, triglycerides ≥200 mg/dL, LDL-C ≥160 mg/dL, and HDL-C<40 mg/dL or a physician’s previous diagnosis of hyperlipidemia. The National Cholesterol Education Program Adult Treatment Panel III (NCEP/ATPIII) Metabolic Syndrome (MetS) criteria were suggested, requiring three of five factors: abdominal obesity as evaluated by sex-specific waist circumference, triglyceride levels, low high-density lipoprotein cholesterol (HDL-C), hypertension, and increased fasting glucose, without the exclusion of diabetes (45). Because the sorts of medications taken by participants vary greatly, we classified the patients’ anti-inflammation therapy into two categories: no (the patients did not take anti-inflammation drugs) and yes (the patients received anti-inflammation drugs). The body mass index (BMI) is the measure of dividing the weight (kg) by the square of the height (m^2^). According to World Health Organization standards, participants’ BMI was classified as<25, 25-29.9, and ≥30 kg/m^2^, corresponding to normal weight, overweight, and obesity, respectively (46).

### Covariates

This investigation included covariates that may impact the relationship between SII and DKD. Demographic parameters included age, gender, race, education level, smoking status, poverty income ratio (PIR), BMI, systolic blood pressure (SBP), and diastolic blood pressure (DBP). Serum creatinine (Scr), blood urea nitrogen (BUN), serum uric acid (SUA), total cholesterol (TC), triglycerides (TG), alanine aminotransferase (ALT), aspartate aminotransferase (AST), and glycohemoglobin were included in the biochemical profile. Health risk factors include hypertension, hyperlipidemia, MetS, atherosclerotic cardiovascular disease, and chronic heart failure. DM-related treatment included the anti-inflammation therapy, metformin use, insulin use, ACEI use, and ARB use. The complete measurement techniques for these variables were easily accessed at www.cdc.gov/nchs/nhanes/.

### Statistical analyses

Given the complex sampling survey, weighted analyses were performed according to the recommendations of the NHANES. The weighted student’s t-test (continuous variables) or weighted chi-square test (categoric variables) were utilized to compare differences in baseline characteristics between the non-DKD and DKD groups in T2DM patients. The receiver operating characteristics curve was used to determine the optimal cutoff value of the SII level. Weighted univariate logistic regression analysis and multivariate logistic regression analysis were used to evaluate the correlation between SII and DKD in different models. In Model 1, confounding variables were not adjusted. In Model 2, age, race, education, and smoking status were adjusted. In Model 3, age, gender, race, PIR, educational levels, smoking status, hypertension, hyperlipidemia, MetS, anti-inflammation therapy, metformin use, insulin use, SBP, DBP, glycohemoglobin, BUN, SUA, TC, TG, ALT, AST, ASCVD, and CHF were adjusted. To determine the potential effect moderators, patients were divided into subgroups based on their age (< 60 or ≥ 60years), sex (male or female), BMI (< 25, 25-29.9 or ≥ 30 kg/m^2^), eGFR (< 60, 60-90 or ≥ 90 ml/min/1.73m^2^), ACR (< 30, 30-300 or ≥ 300 mg/g), hypertension (yes or no), hyperlipidemia (yes or no), anti-inflammation therapy (yes or no), metformin use (yes or no), and insulin use (yes or no). In order to analyze the heterogeneity of correlations among different subgroups, interaction analyses were included as well. Missing values for existing examples of those variables were filled in using the mode for categorical variables or the median for continuous variables. The “nhanesR” package was used to extract and analyze the data. P<0.05 was regarded as statistically significant.

## Results

### Baseline characteristics of participants

A total of 3937 T2DM participants were involved, with an average age of 60.51 years and a gender split of 2040 (51.82%) male patients to 1897 (48.18%) female patients; 1510 (38.35%) participants were categorized as having DKD in our study. Age, poverty income ratio, smoking status, hypertension, hyperlipidemia, anti-inflammation therapy, metformin use, insulin use, ARB use, SBP, DBP, SII, glycohemoglobin, Scr, BUN, SUA, eGFR, TG, ACR, ASCVD, and CHF were significantly different between the two groups (all p<0.05). Sex, race, education levels, MetS, ACEI use, BMI, TC, ALT, and AST did not differ between T2DM patients with and without DKD. 1071 (45.81%) and 524 (36.75%) patients received anti-inflammation therapies in non-DKD and DKD groups, respectively. Among all patients, 63 and 23 patients took ACEI and ARB, respectively. Because the amount of ACEI or ARB use was small and had no significance in statistical analysis, we wouldn’t analyze ACEI or ARB use independently. The clinical and biochemical characteristics of the participants with DKD and non-DKD were shown in **Table 1**.

**Table 1:** Basic characteristics of participants with Type 2 diabetes mellitus (n = 3937) in the NHANES 2011-2018.

Characteristics	Non-DKD (n=2427)	DKD (n=1510)	P-value
Age (years)	56.08 ± 0.39	64.20 ± 0.55	< 0.0001
Gender, %			0.9
Female	47.83 (44.30, 51.36)	48.18 (44.52, 51.84)	
Male	52.17 (48.64, 55.70)	51.82 (48.16, 55.48)	
PIR	2.88 ± 0.06	2.51 ± 0.07	< 0.0001
Race, %			0.34
Mexican American	10.52 (7.76, 13.27)	9.60 (6.84, 12.36)	
Non-Hispanic Black	13.43 (10.80, 16.06)	13.74 (11.06, 16.41)	
Non-Hispanic White	59.07 (54.49, 63.65)	61.51 (57.19, 65.83)	
Other Hispanic	6.81 (5.29, 8.34)	5.58 (4.02, 7.13)	
Other Race	10.17 (8.35, 11.98)	9.57 (7.62, 11.53)	
Education level, %			0.1
Above high school	56.10 (52.29, 59.90)	50.97 (47.07, 54.87)	
High school or GED	24.36 (21.29, 27.42)	25.70 (22.35, 29.04)	
Less than high school	19.53 (16.89, 22.18)	23.17 (20.81,25.53)	
Others	0.01 (0.01, 0.04)	0.16 (0.05, 0.37)	
Smoking status, %			0.001
Former	31.24 (28.30, 34.17)	39.78 (36.69, 42.87)	
Never	52.46 (49.32, 55.59)	46.57 (42.81, 50.32)	
Now	16.31 (14.14, 18.47)	13.65 (11.46, 15.85)	
Hypertension			<0.0001
No	36.18 (33.55, 38.81)	18.35 (15.48, 21.21)	
Yes	63.82 (61.19, 66.45)	81.65 (78.79, 84.52)	
Hyperlipidemia			0.02
No	12.51 (10.71, 14.31)	9.74 (8.03, 11.45)	
Yes	87.49 (85.69, 89.29)	90.26 (88.55, 91.97)	
Metabolic syndrome			0.05
No	39.18 (36.21, 42.15)	35.31 (32.40, 38.21)	
Yes	60.82 (57.85, 63.79)	64.69 (61.79, 67.60)	
Anti-inflammation therapy, %			< 0.001
No	54.19 (51.23, 57.14)	63.25 (59.92, 66.58)	
Yes	45.81 (42.86, 48.77)	36.75 (33.42, 40.08)	
Metformin use, %			< 0.001
No	59.13 (56.35,61.90)	68.96 (65.43,72.49)	
Yes	40.87 (38.10,43.65)	31.04 (27.51,34.57)	
Insulin use, %			< 0.0001
No	93.31 (91.78,94.84)	85.79 (82.95,88.63)	
Yes	6.69 (5.16, 8.22)	14.21 (11.37,17.05)	
ACEI use, %			0.18
No	98.17 (97.43,98.92)	98.83 (98.14,99.53)	
Yes	1.83 (1.08,2.57)	1.17 (0.47,1.86)	
ARB use, %			0.01
No	99.40 (98.99,99.80)	99.78 (99.66,99.89)	
Yes	0.60 (0.20,1.01)	0.22 (0.11,0.34)	
BMI (kg/m^2^)	33.13 ± 0.25	33.06 ± 0.30	0.86
SBP (mmHg)	126.84 ± 0.49	135.79 ± 0.80	< 0.0001
DBP (mmHg)	71.44 ± 0.38	69.41 ± 0.54	0.002
SII	546.42 ± 10.13	634.14 ± 13.43	< 0.0001
Glycohemoglobin (%)	6.95 ± 0.04	7.50 ± 0.06	< 0.0001
Serum creatinine (µmol/L)	73.32 ± 0.51	105.58 ± 2.72	< 0.0001
Blood urea nitrogen (mmol/L)	5.08 ± 0.06	7.05 ± 0.13	< 0.0001
Serum uric acid (µmol/L)	323.74 ± 2.48	364.53 ± 3.40	< 0.0001
eGFR (ml/min/1.73m^2^)	92.59 ± 0.50	69.70 ± 1.13	< 0.0001
TC (mmol/L)	4.75 ± 0.04	4.74 ± 0.04	0.78
TG (mmol/L)	2.12 ± 0.05	2.42 ± 0.07	< 0.001
ALT (IU/L)	28.07 ± 0.60	26.39 ± 1.28	0.23
AST (IU/L)	26.26 ± 0.52	26.05 ± 0.77	0.82
Albumin, urine (mg/L)	11.75± 0.24	303.12 ± 26.90	< 0.0001
Creatinine, urine (mg/dl)	119.32 ± 2.53	112.66 ± 2.27	0.06
ACR (mg/g)	10.09± 0.15	314.81 ± 27.65	< 0.0001
ASCVD, %			< 0.0001
No	83.79 (81.61, 85.97)	70.05 (66.62, 73.48)	
Yes	16.21 (14.03, 18.39)	29.95 (26.52, 33.38)	
CHF, %			< 0.0001
No	95.71 (94.59, 96.84)	86.56 (84.24, 88.88)	
Yes	4.29 (3.16, 5.41)	13.44 (11.12, 15.76)	

Mean ± SD was for continuous variables. The percentage (95% confidence interval) was for categorical variables.

NHANES, National Health and Nutrition Examination Survey; DKD, diabetic kidney disease; PIR, poverty income ratio; GED, general educational development; ACEI, angiotensin converting enzyme inhibitor; ARB, angiotensin receptor blocker; BMI, body mass index; SBP, systolic blood pressure; DBP, diastolic blood pressure; SII, systemic immune-inflammation index; eGFR, estimated glomerular filtration rate; TC, total cholesterol; TG, triglycerides; ALT, alanine aminotransferase; AST, aspartate aminotransferase; ACR, albumin: creatinine ratio; ASCVD, atherosclerotic cardiovascular disease; CHF, chronic heart failure.

### SII is associated with increased likelihood of DKD

We converted SII from a continuous variable to a categorical variable and created a number of models to evaluate the independent effects of SII with and without DKD. **Table 2** shows that higher SII levels were associated with a higher likelihood of DKD after controlling for a number of confounding variables. This relationship was significant both in our basic model (OR = 1.538; 95% CI, 1.287-1.837, p< 0.0001) and in our model with the fewest adjustments (OR = 1.58; 95% CI, 1.28-1.96, p< 0.0001). The correlation between SII and DKD remained positive in the fully corrected model (OR = 1.42, 95% CI: 1.10-1.83, P = 0.01). According to univariate logistic analysis, SII was a risk factor for DKD (OR = 1.001, 95% CI: 1.000–1.001, p< 0.001, **Table 3**). Age, PIR, smoking status, hypertension, hyperlipidemia, MetS, anti-inflammation therapy, metformin use, insulin use, SBP, DBP, glycohemoglobin, Scr, BUN, SUA, eGFR, TG, ACR, ASCVD, and CHF remained significantly associated with the odds of DKD in weighted univariate analysis (p< 0.05, **Table 3**).

**Table 2:** Association between SII and DKD in patients with Type 2 diabetes mellitus.

SII	OR	95% CI	P
Model 1^a^			
< 445.21	Reference		
≥ 445.21	1.538	1.538(1.287, 1.837)	< 0.0001
Model 2^b^			
< 445.21	Reference		
≥445.21	1.58	1.58(1.28, 1.96)	< 0.0001
Model 3^c^			
< 445.21	Reference		
≥445.21	1.42	1.42(1.10, 1.83)	0.01

a Model 1 did not adjust for any confounding factors.

b Model 2 adjusted for age, race, education, and smoking status.

c Model 3 adjusted for age, gender, poverty income ratio, race, education levels, smoking status, Hypertension, Hyperlipidemia, Metabolic syndrome, anti-inflammation therapy, metformin use, insulin use, BMI, SBP, DBP, glycohemoglobin, serum creatinine, blood urea nitrogen, serum uric acid, eGFR, TC, TG, ALT, AST, ASCVD, and CHF.

SII, systemic immune-inﬂammation index; DKD, diabetic kidney disease; OR, odds ratio; CI, confidence interval.

**Table 3:** Univariate logistic regression models of DKD.

Variables	OR (95%CI)	P-value
SII	1.001 (1.000, 1.001)	<0.001
Age (years)	1.048 (1.040, 1.056)	<0.0001
Gender (versus female)		
Male	0.986 (0.793, 1.226)	0.898
PIR	0.868 (0.813, 0.927)	<0.0001
Race (versus Mexican American)		
Non-Hispanic Black	1.121 (0.891, 1.409)	0.324
Non-Hispanic White	1.141 (0.917, 1.419)	0.232
Other Hispanic	0.896 (0.667, 1.203)	0.46
Other Races	1.031 (0.764, 1.393)	0.837
Education level (versus Above high school)		
High school or GED	1.161 (0.849, 1.589)	0.345
Less than high school	1.305 (1.027, 1.660)	0.03
Others	14.110 (0.515, 386.948)	0.115
Smoking status (versus Former)		
Never	0.697 (0.556, 0.873)	0.002
Now	0.657 (0.521, 0.830)	<0.001
Hypertension (versus No)		
Yes	2.523 (2.049, 3.107)	<0.0001
Hyperlipidemia (versus No)		
Yes	1.325 (1.041, 1.686)	0.023
Metabolic syndrome (versus No)		
Yes	1.180 (1.002, 1.391)	0.048
Anti-inflammation therapy (versus No)		
Yes	0.687 (0.567, 0.832)	<0.001
Metformin use (versus No)		
Yes	0.651 (0.527, 0.804)	<0.001
Insulin use (versus No)		
Yes	2.312 (1.610, 3.320)	<0.0001
BMI (kg/m^2^)	0.999 (0.987, 1.011)	0.86
SBP (mmHg)	1.027 (1.021, 1.033)	<0.0001
DBP (mmHg)	0.987 (0.978, 0.995)	0.003
Glycohemoglobin (%)	1.221 (1.164, 1.280)	<0.0001
Serum creatinine (µmol/L)	1.040 (1.035, 1.045)	<0.0001
Blood urea nitrogen (mmol/L)	1.411 (1.347, 1.478)	<0.0001
Serum uric acid (µmol/L)	1.005 (1.004, 1.006)	<0.0001
eGFR (ml/min/1.73m^2^)	0.956 (0.951, 0.962)	<0.0001
TC (mmol/L)	0.990 (0.922, 1.063)	0.777
TG (mmol/L)	1.093 (1.031, 1.157)	0.003
ALT (IU/L)	0.997 (0.987, 1.007)	0.525
AST (IU/L)	0.999 (0.995, 1.004)	0.823
Albumin, urine (mg/L)	1.069 (1.062, 1.076)	<0.0001
Creatinine, urine (mg/dl)	0.999 (0.997, 1.000)	0.071
ACR (mg/g)	1.127 (1.115, 1.139)	<0.0001
ASCVD (versus No)		
Yes	2.210 (1.747, 2.796)	<0.0001
CHF (versus No)		
Yes	3.468 (2.526, 4.760)	<0.0001

For continuous variables and categorical variables, the unit and the reference group are presented beside the variables separately.

OR, odds ratio; CI, confidence interval; DKD, diabetic kidney disease; PIR, poverty income ratio; GED, general educational development; BMI, body mass index; SBP, systolic blood pressure; DBP, diastolic blood pressure; SII, systemic immune-inflammation index; eGFR, estimated glomerular filtration rate; TC, total cholesterol; TG, triglycerides; ALT, alanine aminotransferase; AST, aspartate aminotransferase; ACR, albumin: creatinine ratio; ASCVD, atherosclerotic cardiovascular disease; CHF, chronic heart failure.

Participants who did not complete high school had 30% higher likehood of DKD than those who completed high school (p=0.03). Compared with T2DM patients who had a former smoking history, never and now smoking patients had 30.3% and 34.3% lower likelihood of DKD, respectively (all p< 0.05). Compared with non-hypertension, non-hyperlipidemia, non-MetS, not using insulin participants, non-ASCVD, and non-CHF patients, hypertension, hyperlipidemia, MetS, using insulin, ASCVD, and CHF patients, had 1.523 times, 32.5%, 18%, 1.3 times, 1.11 times, and 2.468 times higher odds of DKD, respectively (all p< 0.05). Compared with T2DM patients who did not receive anti-inflammation therapy and take metformin, patients who received anti-inflammation therapy and took metformin had 31.3% and 34.9% lower likelihood of DKD, respectively (all p< 0.001). Per unit increase in SBP, glycohemoglobin, BUN, serum uric acid, and TG, the odds of DKD were elevated by 2.7% (p< 0.0001), 22.1% (p< 0.0001), 41.1% (p< 0.0001), 0.5% (p< 0.001), and 9.3% (p = 0.003), respectively.

### Subgroup analysis

**Figure 2:**
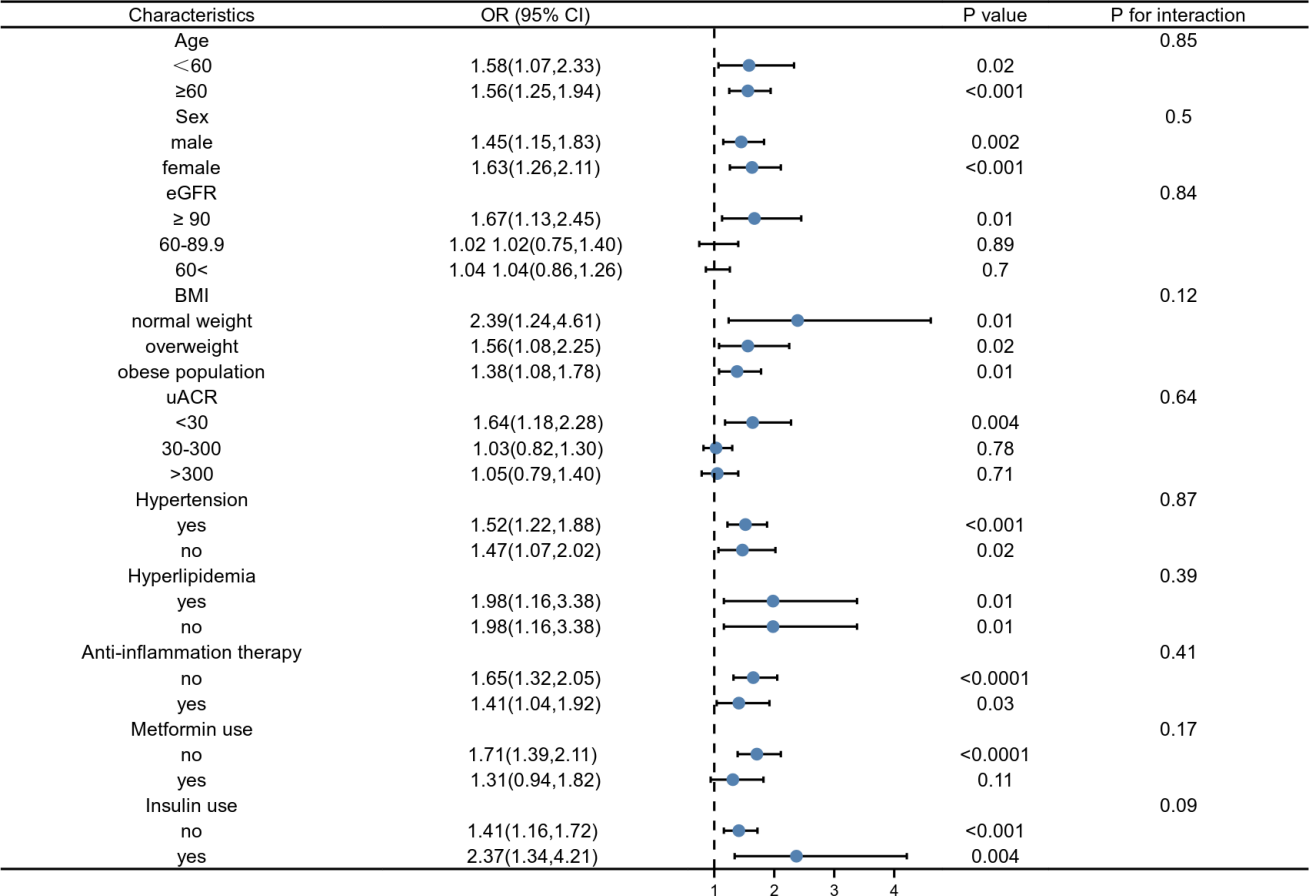
Subgroup analysis for the association between SII and DKD.

The results of our subgroup analysis showed that there were inconsistent associations between SII levels and DKD (**Figure 2**). For the subgroup stratified by age, gender, BMI, hypertension, hyperlipidemia, anti-inflammation therapy, and insulin use, a significant relationship of SII with DKD was detected in each subgroup (all p< 0.05) (**Figure 2**). As for the subgroup stratified by eGFR and ACR, the association with statistical significance was only observed in those with eGFR ≥90 ml/min/1.73m^2^ (p=0.01) and ACR< 30 mg/g. For participants who used metformin, a positive association between SII and DKD was also observed, while this association did not meet the statistical significance (OR = 1.31; 95%CI: 0.94–1.82, p = 0.11). The interaction test showed that there was no significant difference among each stratification in the association between SII and DKD, indicating that there was no significant dependence of age, gender, eGFR, ACR, BMI, hypertension, hyperlipidemia, anti-inflammation therapy, metformin use, and insulin use on this positive association (all p for the interaction > 0.05, **Figure 2**).

## Discussion

The incidence of diabetes worldwide has considerably increased with the growth of the global economy. T2DM prevalence continues to expand globally, with 537 million individuals aged 20 to 79 years suffering from DM in 2021 (47). According to the International Diabetes Federation Diabetes Atlas, the number is expected to increase to 783 million by 2045 (47). DKD, as a major healthcare challenge, affects more than 40% of the >29 million people with T2DM in the United States (48). DKD, a leading cause of ESKD, which calls for dialysis or renal transplantation as treatments, is also correlated to significantly higher cardiovascular morbidity and mortality. Therefore, a thorough awareness of the potential factors that increase the development of lesions can reduce diabetes complications and enhance patients’ quality of life.

To the best of our knowledge, this is the first study to show the relationship between SII and DKD in participants with T2DM. We discovered that SII levels were clearly greater in DKD patients than in non-DKD patients. According to subgroup analysis and an interaction test, this connection was consistent in a diverse demographic setting. Furthermore, high SII levels were associated with an increased likelihood of DKD, providing concrete evidence for further clinical and basic investigation.

Numerous studies have shown the vital role of chronic inflammation in the development of DKD in diabetic individuals. The genome-wide transcriptome analysis revealed a high prevalence of inflammatory signaling pathways in DKD (49). DKD, as the most significant target of microvascular injury in DM, is connected to both systemic and local kidney inflammation with the involvement of essential inflammatory cells and molecules. As simple indicators of inflammation, neutrophils (21), monocytes, lymphocytes (50), and platelet cells (51) have previously been found to be related to the development of DKD in patients with diabetes (52, 53). Platelets are an atypical first-line inflammatory biomarker that may attach to leukocytes and endothelial cells, altering the activity of these cells’ inflammatory components. Many cytokines originate from activated platelets and modulate platelet function in the pathogenesis of DKD, such as IL-1 and IL-6 (54). The main components of the WBC are neutrophils and lymphocytes, which mediate adaptive and innate immunity, respectively. The majority of white blood cells are neutrophils, which play a crucial role in the initiation and regulation of inflammatory processes. Neutrophils also release neutrophil elastase (NE), which plays a role in chronic inflammation. Patients with increased neutrophil activity release reactive oxygen species and NE, which may directly cause renal cell damage, contributing to the continuing development of DKD in T2DM patients. A recent study found that neutrophil count was the most reliable independent risk factor for CKD in both cross-sectional and cohort studies (21). Lymphocytes are a component of leukocytes that mediate adaptive immunity and have a role in innate immunity. Lymphocytes are inflammatory mediators that do have regulatory or protective functions. However, prior research indicated that neither the lymphocyte count nor the neutrophil-to-lymphocyte ratio (NLR) were independent risk factors for DKD in diabetic patients (21, 52).

SII was calculated by counting three kinds of circulatory immune cells: neutrophils, lymphocytes, and platelets (22). The SII level can provide more clinical information than one or two kinds of peripheral blood. Patients with high SII levels often have thrombocytosis, neutrophilia, or lymphopenia (23). The SII level reflects the inflammatory reactions and may be a useful diagnostic biomarker for systemic inflammatory activity. Many studies have demonstrated that SII has remarkable predictive ability (32). In the cross-sectional study, our research revealed that peripheral blood SII was associated with an increased likelihood of DKD.

Some drugs seem to have a beneficial effect on patients with T2DM and CKD. There is a lot of evidence that metformin has anti-hyperglycemic and reno-protective properties that might relate to the mechanisms of anti-inflammation (35). ACEI and ARB also have anti-inflammatory properties in both the general population and in those with CKD (37, 55). Unfortunately, there are fewer patients using ACEI or ARB in our study, which may be related to sampling bias and systematic bias in the process of data extraction. On the contrary, insulin therapy can worsen IR and cause increased inflammation. As expected, our univariate logistic analysis demonstrated that participants who used insulin showed a higher risk of DKD than those who did not use insulin. In addition, the interaction analyses demonstrated that neither anti-inflammation therapy & metformin use, nor insulin use had significant interaction with SII on DKD, ensuring our conclusion’s credibility. To sum up, our study demonstrated that SII may have a significant association with DKD independently.

In our study, age, PIR, smoking status, hypertension, hyperlipidemia, MetS, BUN, SUA, TG, ASCVD, and CHF were all identified as risk factors for DKD in individuals with T2DM, which is consistent with prior research (56). In an animal investigation, Sembach et al. observed no or only minor gender differences in functional and structural alterations throughout DKD development, which is consistent with our results (57). It is apparent that the development of DKD is a complicated process involving multiple components.

SII was a widely available method with a non-intrusive methodology, simple accessibility, and low cost. The potential for therapeutic use is indeed positive. Our research has its own advantages. First, the sample size is sufficient, and the sample selection is representative. Second, to get more trustworthy results, we adjusted for confounding variables. However, the study’s shortcomings call for cautious interpretation of the findings. First, the cross-sectional study design precluded us from establishing a causal association. Secondly, despite the fact that we made adjustments for several relevant confounders, we were incapable of totally ruling out the impact of additional potential confounding variables. Thirdly, while SII is simple to quantify in clinical practice, the loss of neutrophils, lymphocytes, and platelet counts is frequent and may contribute to selection bias. Finally, while we adjusted for a number of possible confounders, additional unmeasured confounders may exist, such as duration of DM, insulin type, drug dosage, and medications, to affect the conclusion. Therefore, future research with a larger number of participants and more accurate measurement is still necessary to define the causal relationship.

## Conclusion

In our study, we proved that SII levels were significantly higher in T2DM patients with DKD than in the subjects with non-DKD, and SII levels are associated with an increased likelihood of DKD in T2DM patients, which may have predictive and diagnostic significance in clinical practice. To validate our findings, further extensive prospective investigations are still required.

## Data availability statement

The publicly available datasets presented in this study can be found in online repositories. These data can be found here: https://www.cdc.gov/nchs/nhanes/.

## Ethics statement

The studies involving human participants were reviewed and approved by National Center for Health Statistics Research Ethics Review Board. The patients/participants provided their written informed consent to participate in this study.

## Author contributions

WG and LS put forward the conception and design of the study. WG and YcS collected and analyzed the data. YS, HD, YC, QY, and HF made the tables and figures. All the authors drafted and revised the paper. All the authors contributed to the article and approved the final version of the manuscript.

## References

[1] Fu H Liu S Bastacky SI Wang X Tian XJ Zhou D. Diabetic kidney diseases revisited: A new perspective for a new era. Mol Metab (2019) 30:250–63. doi: 10.1016/j.molmet.2019.10.005 PMC6838932 31767176

[2] Tuttle KR Bakris GL Bilous RW Chiang JL de Boer IH Goldstein-Fuchs J. Diabetic kidney disease: A report from an ada consensus conference. Am J Kidney Dis (2014) 64(4):510–33. doi: 10.1053/j.ajkd.2014.08.001 25257325

[3] Martínez-Castelao A Navarro-González JF Górriz JL de Alvaro F. The concept and the epidemiology of diabetic nephropathy have changed in recent years. J Clin Med (2015) 4(6):1207–16. doi: 10.3390/jcm4061207 PMC4484995 26239554

[4] Miller RG Mahajan HD Costacou T Sekikawa A Anderson SJ Orchard TJ. A contemporary estimate of total mortality and cardiovascular disease risk in young adults with type 1 diabetes: The Pittsburgh epidemiology of diabetes complications study. Diabetes Care (2016) 39(12):2296–303. doi: 10.2337/dc16-1162 PMC5127232 27654986

[5] Leon-Abarca JA Memon RS Rehan B Iftikhar M Chatterjee A. The impact of covid-19 in diabetic kidney disease and chronic kidney disease: A population-based study. Acta BioMed (2020) 91(4):e2020161. doi: 10.23750/abm.v91i4.10380 33525210 PMC7927495

[6] Galiero R Simeon V Loffredo G Caturano A Rinaldi L Vetrano E. Association between renal function at admission and covid-19 in-hospital mortality in southern Italy: Findings from the prospective multicenter Italian covoca study. J Clin Med (2022) 11(20):1–15. doi: 10.3390/jcm11206121 PMC9604778 36294442

[7] Forbes JM Thorburn DR. Mitochondrial dysfunction in diabetic kidney disease. Nat Rev Nephrol (2018) 14(5):291–312. doi: 10.1038/nrneph.2018.9 29456246

[8] Mosterd CM Kanbay M van den Born BJH van Raalte DH Rampanelli E. Intestinal microbiota and diabetic kidney diseases: The role of microbiota and derived metabolites inmodulation of renal inflammation and disease progression. Best Pract Res Clin Endocrinol Metab (2021) 35(3):101484. doi: 10.1016/j.beem.2021.101484 33546983

[9] Pérez-Morales RE Del Pino MD Valdivielso JM Ortiz A Mora-Fernández C Navarro-González JF. Inflammation in diabetic kidney disease. Nephron (2019) 143(1):12–6. doi: 10.1159/000493278 30273931

[10] Liu C Yang M Li L Luo S Yang J Li C. A glimpse of inflammation and anti-inflammation therapy in diabetic kidney disease. Front Physiol (2022) 13:909569. doi: 10.3389/fphys.2022.909569 35874522 PMC9298824

[11] Acierno C Caturano A Pafundi PC Nevola R Adinolfi LE Sasso FC. Nonalcoholic fatty liver disease and type 2 diabetes: Pathophysiological mechanisms shared between the two faces of the same coin. Explor Med (2020) 1(5):287–306. doi: 10.37349/emed.2020.00019

[12] Wilson PC Wu H Kirita Y Uchimura K Ledru N Rennke HG. The single-cell transcriptomic landscape of early human diabetic nephropathy. Proc Natl Acad Sci U.S.A. (2019) 116(39):19619–25. doi: 10.1073/pnas.1908706116 PMC6765272 31506348

[13] Levin A Reznichenko A Witasp A Liu P Greasley PJ Sorrentino A. Novel insights into the disease transcriptome of human diabetic glomeruli and tubulointerstitium. Nephrol Dial Transplant (2020) 35(12):2059–72. doi: 10.1093/ndt/gfaa121 PMC7716805 32853351

[14] Donath MY. Targeting inflammation in the treatment of type 2 diabetes: Time to start. Nat Rev Drug Discovery (2014) 13(6):465–76. doi: 10.1038/nrd4275 24854413

[15] Liu Q Jiang CY Chen BX Zhao W Meng D. The association between high-sensitivity c-reactive protein concentration and diabetic nephropathy: A meta-analysis. Eur Rev Med Pharmacol Sci (2015) 19(23):4558–68. 26698253

[16] Tang M Cao H Wei XH Zhen Q Liu F Wang YF. Association between high-sensitivity c-reactive protein and diabetic kidney disease in patients with type 2 diabetes mellitus. Front Endocrinol (Lausanne) (2022) 13:885516. doi: 10.3389/fendo.2022.885516 35784528 PMC9245013

[17] Shankar A Sun L Klein BE Lee KE Muntner P Nieto FJ. Markers of inflammation predict the long-term risk of developing chronic kidney disease: A population-based cohort study. Kidney Int (2011) 80(11):1231–8. doi: 10.1038/ki.2011.283 PMC3260339 21866089

[18] Sun Y Jiang L Shao X. Predictive value of procalcitonin for diagnosis of infections in patients with chronic kidney disease: A comparison with traditional inflammatory markers c-reactive protein, white blood cell count, and neutrophil percentage. Int Urol Nephrol (2017) 49(12):2205–16. doi: 10.1007/s11255-017-1710-z 28956241

[19] Dávila-Collado R Jarquín-Durán O Solís-Vallejo A Nguyen MA Espinoza JL. Elevated monocyte to lymphocyte ratio and increased mortality among patients with chronic kidney disease hospitalized for covid-19. J Pers Med (2021) 11(3):1–12. doi: 10.3390/jpm11030224 PMC8004261 33809858

[20] Zeng M Liu Y Liu F Peng Y Sun L Xiao L. J-Shaped association of platelet-to-Lymphocyte ratio with 5-year mortality among patients with chronic kidney disease in a prospective cohort study. Int Urol Nephrol (2020) 52(10):1943–57. doi: 10.1007/s11255-020-02548-1 32661620

[21] Zhang R Chen J Xiong Y Wang L Huang X Sun T. Increased neutrophil count is associated with the development of chronic kidney disease in patients with diabetes. J Diabetes (2022) 14(7):442–54. doi: 10.1111/1753-0407.13292 PMC9310049 35789114

[22] Hu B Yang XR Xu Y Sun YF Sun C Guo W. Systemic immune-inflammation index predicts prognosis of patients after curative resection for hepatocellular carcinoma. Clin Cancer Res (2014) 20(23):6212–22. doi: 10.1158/1078-0432.Ccr-14-0442 25271081

[23] Hong X Cui B Wang M Yang Z Wang L Xu Q. Systemic immune-inflammation index, based on platelet counts and neutrophil-lymphocyte ratio, is useful for predicting prognosis in small cell lung cancer. Tohoku J Exp Med (2015) 236(4):297–304. doi: 10.1620/tjem.236.297 26250537

[24] Miao Y Yan Q Li S Li B Feng Y. Neutrophil to lymphocyte ratio and platelet to lymphocyte ratio are predictive of chemotherapeutic response and prognosis in epithelial ovarian cancer patients treated with platinum-based chemotherapy. Cancer biomark (2016) 17(1):33–40. doi: 10.3233/cbm-160614 27314290 PMC13020469

[25] Geng Y Shao Y Zhu D Zheng X Zhou Q Zhou W. Systemic immune-inflammation index predicts prognosis of patients with esophageal squamous cell carcinoma: A propensity score-matched analysis. Sci Rep (2016) 6:39482. doi: 10.1038/srep39482 28000729 PMC5175190

[26] Chen JH Zhai ET Yuan YJ Wu KM Xu JB Peng JJ. Systemic immune-inflammation index for predicting prognosis of colorectal cancer. World J Gastroenterol (2017) 23(34):6261–72. doi: 10.3748/wjg.v23.i34.6261 PMC5603492 28974892

[27] Huang H Liu Q Zhu L Zhang Y Lu X Wu Y. Prognostic value of preoperative systemic immune-inflammation index in patients with cervical cancer. Sci Rep (2019) 9(1):3284. doi: 10.1038/s41598-019-39150-0 30824727 PMC6397230

[28] Ran Y Wu QN Long YJ Li Q Wu J Da JJ. Association of systemic immune-inflammation index with protein-energy wasting and prognosis in patients on maintenance hemodialysis. Zhonghua Yi Xue Za Zhi (2021) 101(28):2223–7. doi: 10.3760/cma.j.cn112137-20210220-00445 34333935

[29] Wang J Zhou D Dai Z Li X. Association between systemic immune-inflammation index and diabetic depression. Clin Interv Aging (2021) 16:97–105. doi: 10.2147/cia.S285000 33469277 PMC7810592

[30] Xie Y Zhuang T Ping Y Zhang Y Wang X Yu P. Elevated systemic immune inflammation index level is associated with disease activity in ulcerative colitis patients. Clin Chim Acta (2021) 517:122–6. doi: 10.1016/j.cca.2021.02.016 33662359

[31] Zhang Z Chen Z. Higher systemic immune-inflammation index is associated with higher likelihood of peripheral arterial disease. Ann Vasc Surg (2022) 84:322–6. doi: 10.1016/j.avsg.2021.12.011 34954036

[32] Qin Z Li H Wang L Geng J Yang Q Su B. Systemic immune-inflammation index is associated with increased urinary albumin excretion: A population-based study. Front Immunol (2022) 13:863640. doi: 10.3389/fimmu.2022.863640 35386695 PMC8977553

[33] Li Y Liu M Cui Y Zhu Z Chen J Zeng F. Increased risk of testosterone deficiency is associated with the systemic immune-inflammation index: A population-based cohort study. Front Endocrinol (Lausanne) (2022) 13:974773. doi: 10.3389/fendo.2022.974773 36051392 PMC9424499

[34] Tang Y Peng B Liu J Liu Z Xia Y Geng B. Systemic immune-inflammation index and bone mineral density in postmenopausal women: A cross-sectional study of the national health and nutrition examination survey (Nhanes) 2007-2018. Front Immunol (2022) 13:975400. doi: 10.3389/fimmu.2022.975400 36159805 PMC9493473

[35] Salvatore T Pafundi PC Morgillo F Di Liello R Galiero R Nevola R. Metformin: An old drug against old age and associated morbidities. Diabetes Res Clin Pract (2020) 160:108025. doi: 10.1016/j.diabres.2020.108025 31954752

[36] Salvatore T Galiero R Caturano A Vetrano E Rinaldi L Coviello F. Effects of metformin in heart failure: From pathophysiological rationale to clinical evidence. Biomolecules (2021) 11(12):1–23. doi: 10.3390/biom11121834 PMC8698925 34944478

[37] Sasso FC Carbonara O Persico M Iafusco D Salvatore T D'Ambrosio R. Irbesartan reduces the albumin excretion rate in microalbuminuric type 2 diabetic patients independently of hypertension: A randomized double-blind placebo-controlled crossover study. Diabetes Care (2002) 25(11):1909–13. doi: 10.2337/diacare.25.11.1909 12401731

[38] Forst T Mathieu C Giorgino F Wheeler DC Papanas N Schmieder RE. New strategies to improve clinical outcomes for diabetic kidney disease. BMC Med (2022) 20(1):337. doi: 10.1186/s12916-022-02539-2 36210442 PMC9548386

[39] Salvatore T Galiero R Caturano A Rinaldi L Di Martino A Albanese G. An overview of the cardiorenal protective mechanisms of Sglt2 inhibitors. Int J Mol Sci (2022) 23(7):1–44. doi: 10.3390/ijms23073651 PMC8998569 35409011

[40] Mendez CE Walker RJ Eiler CR Mishriky BM Egede LE. Insulin therapy in patients with type 2 diabetes and high insulin resistance is associated with increased risk of complications and mortality. Postgrad Med (2019) 131(6):376–82. doi: 10.1080/00325481.2019.1643635 PMC7052790 31311382

[41] Pradhan AD Everett BM Cook NR Rifai N Ridker PM. Effects of initiating insulin and metformin on glycemic control and inflammatory biomarkers among patients with type 2 diabetes: The lancet randomized trial. Jama (2009) 302(11):1186–94. doi: 10.1001/jama.2009.1347 19755697

[42] Levey AS Stevens LA Schmid CH Zhang YL Castro AF 3rd Feldman HI. A new equation to estimate glomerular filtration rate. Ann Intern Med (2009) 150(9):604–12. doi: 10.7326/0003-4819-150-9-200905050-00006 PMC2763564 19414839

[43] de Boer IH Rue TC Hall YN Heagerty PJ Weiss NS Himmelfarb J. Temporal trends in the prevalence of diabetic kidney disease in the united states. Jama (2011) 305(24):2532–9. doi: 10.1001/jama.2011.861 PMC3731378 21693741

[44] Unger T Borghi C Charchar F Khan NA Poulter NR Prabhakaran D. 2020 International society of hypertension global hypertension practice guidelines. Hypertension (2020) 75(6):1334–57. doi: 10.1161/hypertensionaha.120.15026 32370572

[45] Grundy SM Becker D Clark LT Cooper RS Denke MA Howard WmJ. Executive summary of the third report of the national cholesterol education program (Ncep) expert panel on detection, evaluation, and treatment of high blood cholesterol in adults (Adult treatment panel iii). Jama (2001) 285(19):2486–97. doi: 10.1001/jama.285.19.2486 11368702

[46] Appropriate body-mass index for Asian populations and its implications for policy and intervention strategies. Lancet (2004) 363(9403):157–63. doi: 10.1016/s0140-6736(03)15268-3 14726171

[47] Sun H Saeedi P Karuranga S Pinkepank M Ogurtsova K Duncan BB. Idf diabetes atlas: Global, regional and country-level diabetes prevalence estimates for 2021 and projections for 2045. Diabetes Res Clin Pract (2022) 183:109119. doi: 10.1016/j.diabres.2021.109119 34879977 PMC11057359

[48] Bailey RA Wang Y Zhu V Rupnow MF. Chronic kidney disease in us adults with type 2 diabetes: An updated national estimate of prevalence based on kidney disease: Improving global outcomes (Kdigo) staging. BMC Res Notes (2014) 7:415. doi: 10.1186/1756-0500-7-415 24990184 PMC4091951

[49] Woroniecka KI Park AS Mohtat D Thomas DB Pullman JM Susztak K. Transcriptome analysis of human diabetic kidney disease. Diabetes (2011) 60(9):2354–69. doi: 10.2337/db10-1181 PMC3161334 21752957

[50] Huang W Huang J Liu Q Lin F He Z Zeng Z. Neutrophil-lymphocyte ratio is a reliable predictive marker for early-stage diabetic nephropathy. Clin Endocrinol (Oxf) (2015) 82(2):229–33. doi: 10.1111/cen.12576 25088518

[51] Liu J Liu X Li Y Quan J Wei S An S. The association of neutrophil to lymphocyte ratio, mean platelet volume, and platelet distribution width with diabetic retinopathy and nephropathy: A meta-analysis. Biosci Rep (2018) 38(3):1–18. doi: 10.1042/bsr20180172 PMC6019380 29581246

[52] Chung FM Tsai JC Chang DM Shin SJ Lee YJ. Peripheral total and differential leukocyte count in diabetic nephropathy: The relationship of plasma leptin to leukocytosis. Diabetes Care (2005) 28(7):1710–7. doi: 10.2337/diacare.28.7.1710 15983324

[53] Schmidt MI Duncan BB Sharrett AR Lindberg G Savage PJ Offenbacher S. Markers of inflammation and prediction of diabetes mellitus in adults (Atherosclerosis risk in communities study): A cohort study. Lancet (1999) 353(9165):1649–52. doi: 10.1016/s0140-6736(99)01046-6 10335783

[54] Hojs R Ekart R Bevc S Hojs N. Markers of inflammation and oxidative stress in the development and progression of renal disease in diabetic patients. Nephron (2016) 133(3):159–62. doi: 10.1159/000447434 27344598

[55] Pan M Vasbinder A Anderson E Catalan T Shadid HR Berlin H. Angiotensin-converting enzyme inhibitors, angiotensin ii receptor blockers, and outcomes in patients hospitalized for covid-19. J Am Heart Assoc (2021) 10(24):e023535. doi: 10.1161/jaha.121.023535 34889102 PMC9075226

[56] Bonner R Albajrami O Hudspeth J Upadhyay A. Diabetic kidney disease. Prim Care (2020) 47(4):645–59. doi: 10.1016/j.pop.2020.08.004 33121634

[57] Sembach FE Fink LN Johansen T Boland BB Secher T Thrane ST. Impact of sex on diabetic nephropathy and the renal transcriptome in unx Db/Db C57blks mice. Physiol Rep (2019) 7(24):e14333. doi: 10.14814/phy2.14333 31876119 PMC6930935

